# Shoulder-Pacemaker Treatment Concept for Posterior Positional Functional Shoulder Instability: A Prospective Clinical Trial

**DOI:** 10.1177/0363546520933841

**Published:** 2020-07-15

**Authors:** Philipp Moroder, Fabian Plachel, Heiko Van-Vliet, Christiane Adamczewski, Victor Danzinger

**Affiliations:** † Department of Shoulder and Elbow Surgery, Center for Musculoskeletal Surgery, Charité–Universitaetsmedizin Berlin, Campus Virchow Klinikum, Berlin, Germany; ‡ University of Lausanne, Lausanne, Switzerland; § Rehazentrum Virchow GbR, Berlin, Germany; Investigation performed at the Department for Shoulder and Elbow Surgery, Center for Musculoskeletal Surgery, Campus Virchow, Charité–Universitaetsmedizin Berlin, Berlin, Germany

**Keywords:** functional shoulder instability, posterior shoulder instability, shoulder-pacemaker, EMS treatment, voluntary shoulder instability, rehabilitation, posterior positional functional shoulder instability

## Abstract

**Background:**

Pathological muscle activation patterns of the external rotators and periscapular muscles can result in posterior positional functional shoulder instability (PP-FSI). In several patients, physical therapy and surgical treatment are not successful.

**Purpose:**

The shoulder-pacemaker treatment concept was evaluated prospectively in patients with PP-FSI and previously failed conventional therapy attempt.

**Study Design:**

Case series; Level of evidence, 4.

**Methods:**

A negative selection of 24 consecutive cases of noncontrollable PP-FSI in 16 patients with previously failed conventional therapy were included in this prospective study. The shoulder-pacemaker treatment consisted of an electrical muscle stimulation–based therapy protocol with 9 to 18 one-hour treatment sessions. Two patients were excluded because of nonadherence to the training schedule, leaving a final study cohort of 21 cases in 14 patients. Follow-up included assessment of clinical function, impairment during daily activities and sports, satisfaction, Western Ontario Shoulder Instability Index (WOSI), Rowe score, and Subjective Shoulder Value at 0 weeks, 2 weeks, 4 weeks, 3 months, 6 months, 12 months, and 24 months after intervention.

**Results:**

WOSI, Subjective Shoulder Value, and Rowe score showed a highly significant improvement at all time points of follow-up (*P* < .001). Young age (*P* = .005), low weight (*P* = .019), shoulder activity level (*P* = .003), unilateral affliction (*P* = .046), and higher baseline WOSI score (*P* = .04) were associated with a better treatment effect. Cases with increased glenoid retroversion, posterior scapulohumeral decentering, and dysplastic bony glenoid shape showed a trend toward shorter treatment effect duration. No complications during the intervention or follow-up period were observed.

**Conclusion:**

The shoulder-pacemaker therapy concept is an effective treatment with rapid improvement and sustained outcome over the course of 2 years in patients with noncontrollable PP-FSI with previously failed conventional treatment. Young and more athletic patients with lower weight and unilateral pathology respond best to the treatment.

Shoulder stability is provided by a complex interaction of active and passive anatomic restraints of the shoulder joint.^5^ Several electromyographic studies have highlighted the essential role of the rotator cuff and periscapular muscles for stability of the shoulder joint.^2,12,24,33^ If these muscles do not activate adequately and in a balanced way, a severe type of shoulder instability can result. This type has recently been named functional shoulder instability (FSI),^28^ as opposed to structural shoulder instability, which is caused by structural damage. The estimated prevalence of FSI is 0.5% to 2.6% in a young population.^8^

The most common type of FSI is posterior positional FSI (PP-FSI).^28^ PP-FSI mainly affects teenagers and young adults who experience disabling recurrent posterior subluxations or dislocations during shoulder movement, even in only the midrange of motion, despite the absence of relevant structural defects.^28^ Recent studies identified the cause of PP-FSI as being hypoactivity of the external rotators and posterior deltoid with concurrent hyperactivity of the internal rotators and dysbalance of the periscapular muscles.^1,18,37^ Furthermore, a functional magnetic resonance imaging (MRI) study revealed increased activity within the primary motor cortex, supramarginal gyrus, inferior frontal gyrus, premotor cortex, and middle frontal gyrus during shoulder movement in patients with FSI, indicating increased neural activity similar to early learning of a motor sequence.^15^ Affected patients experience various symptoms, including chronic pain, movement restriction attributed to weakness or blockage, and a persistent feeling of shoulder instability.^28,29^ Extreme limitations during daily activities and sports, as well as “bizarre-looking” dislocations, can lead to stigmatization among peers and emotional stress of the affected patients.^29^

Several classification systems exist to describe this pathology: the ABC classification of posterior shoulder instability refers to PP-FSI as functional posterior shoulder instability (type B1), as opposed to structural posterior shoulder instability (type B2).^32^ The Stanmore classification names shoulder instability caused by nonphysiological muscle activation patterns in general polar type III instability.^26^ Gerber and Nyffeler^10^ defined cases with dislocation and voluntary reduction as type B6.

Electric muscle stimulation (EMS) has been successfully applied in patients affected by hemiplegia and subsequent shoulder instability^19^ after a stroke and had proved more effective than physical therapy or transcutaneous electrical nerve stimulation in randomized controlled trials.^6,40^ In a previous pilot project, EMS was successfully applied to counteract the presumed muscle hypoactivity causing patients to experience PP-FSI.^29^

The aim of the present study was to evaluate this so-called shoulder-pacemaker treatment concept in a larger prospective clinical trial for patients with noncontrollable PP-FSI. The hypothesis was that the shoulder-pacemaker treatment concept would even be effective in patients with previously failed conventional physiotherapy.

## Methods

### Participants

Over the course of 12 months, 16 consecutive patients with noncontrollable PP-FSI presenting to our university outpatient clinic were included in this prospective case series. A total of 8 patients had bilateral PP-FSI. PP-FSI was defined according to a previous publication.^28^ As an inclusion criterion, all participants had received at least 3 months of pathology-targeted physical therapy without success to form a negatively selected study cohort. Exclusion criteria for participation in the study were (1) existing pain syndrome (defined by pain at rest or during motion that is not caused by dislocation and impedes physiotherapeutic training and/or EMS), (2) contraindication to EMS treatment (eg, cardiac pacemaker), and (3) neurological disorders or nerve injuries causing the instability.

Two patients (1 bilateral) were excluded during the intervention phase for nonadherence to the treatment schedule. The follow-up rate for the remaining patients was 100% for all time points. The final study cohort consisted of 21 cases in 14 patients: 7 with unilateral affection and 7 with bilateral. Patient characteristics are outlined in Table 1. While a repetitive microtraumatic cause cannot be excluded in study participants with high shoulder-specific athletic activity level^30^ (29%), most (71%) had atraumatic development of PP-FSI. In regard to previous treatment, 10 shoulders (48%) received pathology-targeted nonoperative treatment for 3 months, 5 (24%) for >3 to 12 months, and 6 (29%) for >1 year. In most cases (86%), guided movement exercises and exercises on machines were attempted for 20 minutes 2 or 3 times per week under the supervision of a physical therapist. Additional treatment approaches included manual therapy (33%), massage (24%), transcutaneous electrical nerve stimulation (19%), or the application of heat/cold therapies (5%) for pain reduction. Furthermore, 4 shoulders (19%) underwent unsuccessful surgical interventions: arthroscopic capsulolabral plication in 2 (10%), not further specified arthroscopic rotator cuff tensioning in 1 (5%), as well as arthroscopic subacromial decompression because of pain in 1 (5%).

**Table 1:** Patient Characteristics^*a*^

		Occupation;		Affected Shoulder;					Glenoid			Posterior Decentering, %	
Sex; Age, y	Height, cm; Weight, kg	Sports Activities	SAL	Time Since Symptom Onset, mo	WOSI	SSV	Rowe	Kim Lesion	Bony Shape	Articular Surface	Retroversion, deg	Gleno- humeral	Scapulo- humeral
F, 17	176, 68	Student	0	R, 3	41	50	40						
				L, 7	10	25	30	N	Flat	Concave	4	41	53
M, 19	180, 70	Student, handball	2	R, 46	37	25	25	N	Flat	Concave	7	63	61
M, 24	187, 78	Military, fitness	2	R, 57	34	30	40	Y	Convex	Convex	30	54	85
				L, 57	46	50	55						
M, 18	171, 85	Student, boxing	2	R, 15	29	50	30	N	Concave	Concave	0	62	58
F, 17	168, 60	Student, judo	1	R, 48	65	70	55						
				L,^*b*^ 48	39	60	40	N	Flat	Concave	5	57	55
M, 33	174, 110	Office	0	R, 130	20	20	15						
				L,^*c*^ 130	28	50	35	N	Concave	Concave	12	59	70
F, 16	167, 57	Student, acrobatics	2	R, 19	28	45	15	N	Flat	Concave	4	49	54
M, 20	187, 90	Student, fitness	2	R, 149	52	85	55						
				L, 149	30	55	60	N	Flat	Concave	10	40	58
M, 22	180, 70	Worker, fitness	2	R, 50	89	100	80						
				L, 50	31	45	40	Y	Flat	Concave	7	43	54
F, 18	165, 60	Student, handball	2	R, 43	29	25	30	N	Concave	Concave	12	51	63
F,^*d*^ 15	180, 63	Student, volleyball	2	R,^*e*^ 19	38	60	40	N	Flat	Concave	3	46	57
M, 18	173, 63	Worker	0	L, 60	17	20	15	Y	Convex	Flat	10	38	55
F, 16	160, 45	Student, dancing	1	R, 11	47	70	75	N	Flat	Concave	0	45	51
				L, 11	82	95	80						
F, 15	165, 50	Student	0	L, 3	45	50	40	N	Concave	Concave	3	32	45

a F, female; L, left; M, male; N, no; R, right; SAL, shoulder activity level; SSV, Subjective Shoulder Value; WOSI, Western Ontario Shoulder Instability Index; Y, yes.

b Reverse Hill-Sachs, γ: 76°.

c Reverse Hill-Sachs, γ: 65°.

d See Figure 1 for clinical and radiological presentation.

e Reverse Hill-Sachs, γ: 79°.

### Clinical Assessment

Before the intervention, all participants received a detailed clinical and radiological examination. All patients showed demonstrable yet uncontrollable unidirectional PP-FSI that repetitively occurred during midrange of motion, even without exertion. Despite the severe instability, most of the patients with PP-FSI had unrestricted range of motion of the affected shoulder.

Of the affected cases, 7 (33%) demonstrated hyperlaxity with a Beighton score^3^ ≥5, as well as a positive sulcus sign,^20^ Gagey test,^9^ and Walch test.^7^ Scapular dyskinesis^21^ was observed in 19 cases (90%), whereas 2 (10%) had a normal scapulothoracic motion. Strength measurements were rendered intolerable owing to the severe grade of instability in most patients.

### Radiological Assessment

All participants revealed unidirectional PP-FSI with subluxation or dislocation and spontaneous reduction under dynamic fluoroscopic assessment (Ziehm 8000; Ziehm Imaging Gmbh) (Figure 1). If bilateral PP-FSI was clinically evident in a patient, only 1 side was analyzed with fluoroscopy to minimize radiation exposure. MRI was obtained from all study participants. In the case of bilateral PP-FSI, only the more unstable side was evaluated with MRI. Image analysis revealed 3 type I Kim lesions^22^ (21%) and 3 noncritical reverse Hill-Sachs lesions (21%) with a mean ± SD gamma angle^31^ of 73° ± 6° (range, 65°-79°) (Table 1).

Bony glenoid shape analysis showed a concave shape in 4 cases (29%), flat shape in 8 (57%), and convex shape in 2 (14%). Glenoid articular surface shape analysis revealed a concave articular surface in 12 cases (86%), flat surface in 1 (7%), and convex surface in 1 (7%). The mean retroversion was 8° ± 7.3° (range, 0°-30°). The mean static posterior glenohumeral decentering^31^ (with 50% representing a centered joint) was 49% ± 9% (range, 32%-63%), and the mean static posterior scapulohumeral decentering^38^ (with 50% representing a centered joint) was 58% ± 10% (range, 45%-85%). No other structural defects or abnormalities were noticed. No signs of instability arthropathy were observed in this patient cohort despite the high frequency of daily instability episodes over long periods.

**Figure 1.**
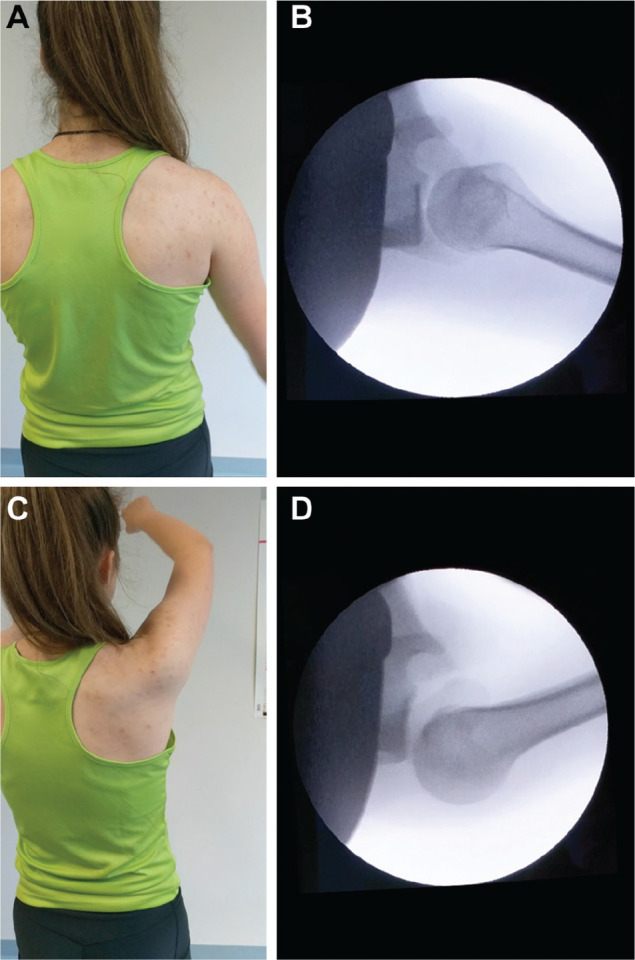
Clinical and radiological presentation of PP-FSI in a young female patient (see Table 1). (A) The neutral position without signs of posterior instability. (B) The corresponding fluoroscopic image with centered humeral head. (C) Changes in the shoulder contour can be observed. (D) The posterior subluxation of the humeral head during motion is revealed.

### Rehabilitation Protocol

**Figure 2.**
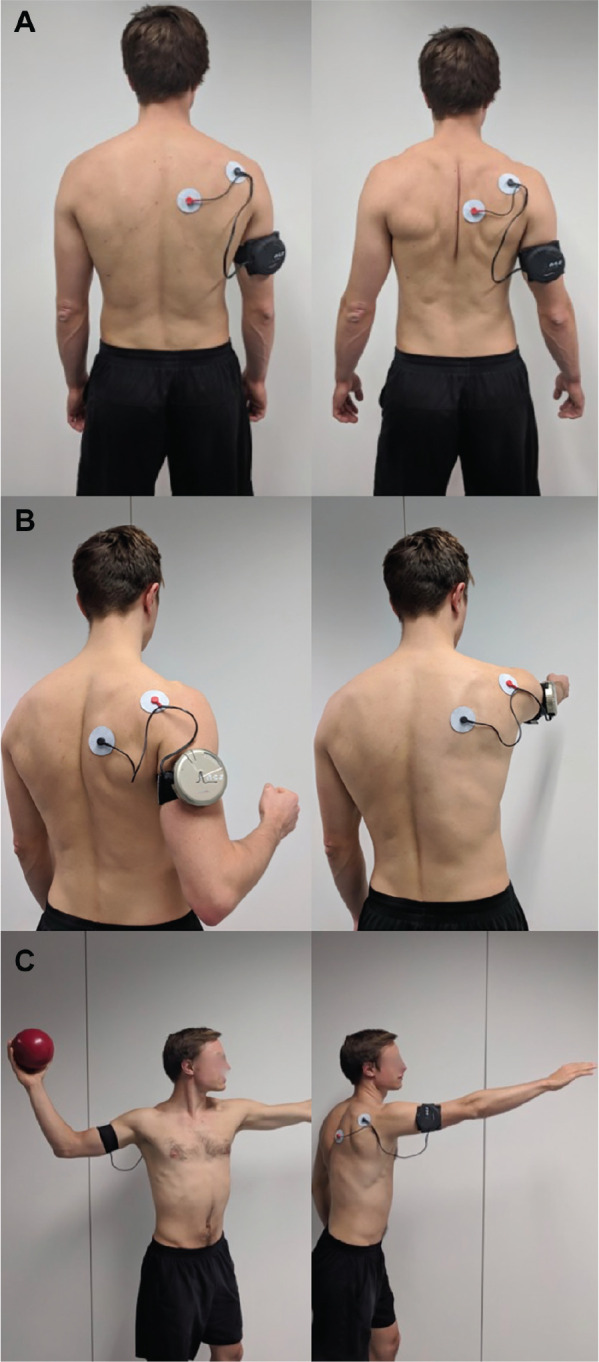
Example of transdermal electric muscle stimulation by means of a dedicated device (Shoulder Pacemaker, NCS Lab) to activate hypoactive muscle groups during (A) concentric, (B) eccentric, and (C) functional training (eg, throwing motion). Concentric exercises aim for activation and tonic contraction of hypoactive muscle groups through maximum tolerable intensity of the device. Eccentric exercises aim for strengthening of hypoactive muscle groups by eccentric exercises against the tonic contraction induced by the electrical stimulus. Functional exercises aim for electrically assisted activation of hypoactive muscles during movement exercises that are intended to restore an instability-free motion of the shoulder joint during complex movements (eg, patient-oriented sports training).

The treatment concept consisted of motion exercises performed while EMS was applied, followed by physical therapy without EMS. Different types of EMS devices were used for this study, ranging from conventional general-purpose devices to more dedicated shoulder-specific EMS devices where muscle stimulation could be automatically guided by the patient’s arm movement. All the devices were battery powered, and all produced symmetric compensated rectangular alternating current with a frequency of 35 Hz, which was applied by means of 2 transdermal electrodes to achieve a tonic contraction of hypoactive external rotators and scapula-retracting muscle groups. The first electrode was applied inferior to the spina scapulae to stimulate the external rotators (infraspinatus, teres minor, and posterior deltoid). The second electrode was positioned medial to the medial border of the scapula to stimulate the scapular retractors (transverse portion of the trapezius and the rhomboids). The EMS devices remained attached throughout the first 30 minutes of the physiotherapeutic treatment to stimulate hypoactive muscle groups during concentric, eccentric, and functional movement exercises (Figure 2). In the following 30 minutes, the stimulated muscle groups were further activated by physiotherapeutic exercises without electrical stimulation. All participants received three 1-hour units of treatment per week for 3 weeks. If the symptoms persisted after a total of 9 units, the treatment was prolonged for additional 3 weeks. Over the course of treatment, the stimulation intensity and exercise difficulty (levels 1-3) were increased (see Appendix, available in the online version of article). No aftercare exercises or lifestyle recommendations were given to the patients after the intervention period.

### Follow-up

Participants were assessed before the treatment intervention and 0 weeks, 2 weeks, 4 weeks, 3 months, 6 months, 12 months, and 24 months after treatment had ended.

Outcome assessment included the Subjective Shoulder Value, Rowe score, and the Western Ontario Shoulder Instability Index (WOSI).^11,23,35^ The reason we chose subjective outcome measurements was the fact that (1) the patients’ perception of their shoulder function is a key factor to determine the success of treatment^36^ and (2) a comprehensive, valid, and reliable objective clinical or radiographic outcome measurement in this highly dynamic pathology is not available.

Further assessment included functional impairment during daily as well as sporting activities, satisfaction with the therapy concept, and overall recommendation of the intervention. Video documentation of the instability mechanism and instability-free function at follow-up was obtained in all patients.

### Ethics

Approval of the ethical committee of the Charité University was obtained (EA2/195/16). Informed consent was obtained from all study participants, including parental consent for minors.

### Statistical Analysis

Descriptive statistics were performed to analyze mean, standard deviation, and range, including frequency counts and percentages when appropriate. All collected data were analyzed for normal distribution with the Kolmogorov-Smirnov test. The Wilcoxon test for paired samples was applied for comparison of baseline and follow-up means, since data were not normally distributed. The Mann-Whitney *U* test was used to compare the WOSI directly after treatment as well as at 1- and 2-year follow-up between unrelated subgroups. Furthermore, to address the effect of the described treatment protocol on shoulder stability, we used either Pearson correlation or Spearman rank order correlation analysis, in which the dependent variable was the WOSI score directly after treatment as well as at 1- and 2-year follow-up. The alpha level was set to .05.

## Results

After the intervention, all participants were able to achieve a stable shoulder motion without signs of posterior subluxations or dislocations. While the majority of patients (79%) successfully finished the training period after 3 weeks of participation, in 3 (21%) the treatment had to be prolonged for another 3 weeks: in 2 with bilateral PP-FSI because of residual posterior subluxation during end range of motion and in 1 because of a sustained minor trauma and treatment setback during the intervention period.

During 2-year follow-up, 1 patient developed atraumatic recurrence of instability 6 months after the treatment had ended, and 1 had a traumatic recurrence 2 weeks after the treatment had ended. A third (pretreatment WOSI, 28%) had an excellent treatment effect for 1 year (WOSI, 80%) and then developed atraumatic recurrence of instability. After completion of the 2-year follow-up (WOSI, 12%), this patient was retrained with the same protocol and regained an excellent outcome (WOSI, 84%). In another case, residual instability during end range of motion was observed at the 3 months’ follow-up but not at the subsequent follow-up time points despite the lack of any further intervention. Finally, in 1 case, a subluxation during a handstand was reported after 7 months, but no further instability episodes were noticed during the remainder of follow-up. Even though the intervention successfully treated the previously noncontrollable instability in most cases, the patients still were able to perform a deliberate, controllable dislocation of their shoulder after intervention.

**Figure 3.**
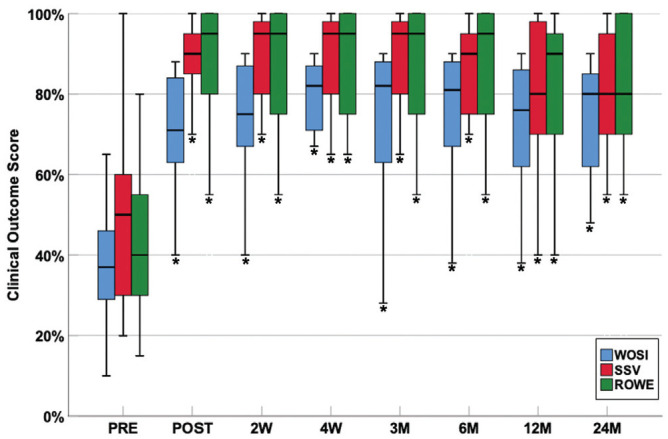
Longitudinal assessment of the clinical outcome scores before the treatment intervention (PRE) and 0 weeks (POST), 2 weeks (2W), 4 weeks (4W), 3 months (3M), 6 months (6M), 12 months (12M), and 24 months (24M) after treatment. *P < .001, vs baseline. Values are presented as median (line), interquartile range (box), and maximum and minimum except outliers and extremes (bars). ROWE, Rowe score; SSV, Subjective Shoulder Value; WOSI, Western Ontario Shoulder Instability Index.

**Figure 4.**
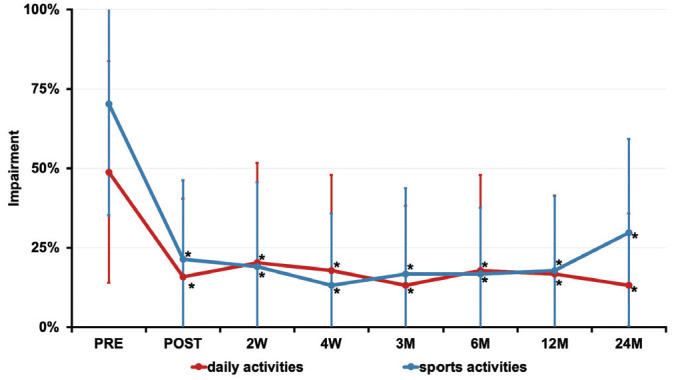
Longitudinal assessment of the functional impairment during daily and sporting activities before the treatment intervention (PRE) and 0 weeks (POST), 2 weeks (2W), 4 weeks (4W), 3 months (3M), 6 months (6M), 12 months (12M), and 24 months (24M) after treatment. *P < .01, vs baseline. Values are presented as mean ± SD.

All clinical outcome scores (WOSI, Subjective Shoulder Value, Rowe score) showed a highly significant improvement (*P* < .001) after the end of the treatment, and the results were sustained over the course of 2 years (Figure 3). Corresponding assessment of functional impairment during sports as well as daily activities also showed a significant reduction (*P* < .01) after the intervention had ended and during the follow-up period (Figure 4).

An association was identified between a better treatment effect and young age (*P* = .005), low weight (*P* = .019), higher shoulder activity level (*P* = .003), higher baseline WOSI score (*P* = .04) (Table 2), and unilateral pathology (*P* = .046) (Table 3).

**Table 2:** Association Between Patient Characteristics Before and After the Intervention: Western Ontario Shoulder Instability Index (WOSI)^*a*^

	Postoperative		1 y		2 y	
	*R*	*P* Value	*R*	*P* Value	*R*	*P* Value
Patient age, y	–0.593	**.005**	–0.528	**.014**	–0.206	.369
Height, cm	–0.186	.419	–0.271	.235	–0.017	.943
Weight, kg	–0.508	**.019**	–0.424	.056	–0.169	.463
Duration of symptoms, mo	–0.366	.103	–0.418	.059	–0.220	.337
Months of failed nonoperative therapy	–0.251	.272	–0.188	.415	–0.175	.447
Sports-activity level, points	0.607	**.003**	0.427	**.005**	0.034	.884
WOSI score before intervention	0.451	**.040**	0.382	.088	0.195	.398
Increased glenoid retroversion, degree	–0.417	.138	–0.715	**.004**	–0.170	.562
Posterior glenohumeral decentering, %	0.200	.492	0.023	.937	0.053	.857
Posterior scapulohumeral decentering, %	–0.316	.270	–0.608	**.021**	–0.132	.652

a Bold indicates *P* < .05.

**Table 3:** Comparison of Western Ontario Shoulder Instability Index Score Between Subgroups^*a*^

	Postoperative		1 y		2 y	
	Mean ± SD, %	*P* Value	Mean ± SD, %	*P* Value	Mean ± SD, %	*P* Value
Sex (n = 21)		.809		.426		.863
Men (n = 11)	69 ± 18		64 ± 24		72 ± 17	
Women (n = 10)	72 ± 12		76 ± 10		68 ± 23	
Occurrence (n = 21)		**.046**		**.031**		.172
Unilateral (n = 7)	80 ± 10		79 ± 19		71 ± 29	
Bilateral (n = 14)	66 ± 15		65 ± 18		70 ± 14	
Hyperlaxity (n = 21)		.913		.535		.585
No (n = 14)	70 ± 16		69 ± 23		72 ± 16	
Yes (n = 7)	70 ± 13		71 ± 7		67 ± 26	
Previous surgery (n = 21)		≥.999		.965		.574
No (n = 17)	71 ± 15		70 ± 20		71 ± 21	
Yes (n = 4)	69 ± 19		70 ± 19		68 ± 14	
Labral lesion (n = 14)		.769		.170		.653
No (n = 11)	71 ± 16		75 ± 15		67 ± 23	
Yes (n = 3)	67 ± 15		49 ± 33		74 ± 20	
Reverse Hill-Sachs lesion (n = 14)		.368		.368		.291
No (n = 11)	72 ± 15		71 ± 23		71 ± 25	
Yes (n = 3)	63 ± 18		63 ± 16		61 ± 3	
Bony glenoid shape (n = 14)		.647		**.044**		.774
Concave (n = 4)	71 ± 21		79 ± 21		79 ± 21	
Flat (n = 8)	73 ± 13		74 ± 12		65 ± 25	
Convex (n = 2)	60 ± 12		30 ± 10		75 ± 16	
Articular surface shape (n = 14)		.525		.088		.506
Concave (n = 12)	72 ± 15		75 ± 15		68 ± 22	
Flat (n = 1)	68 ± 0		38 ± 0		90 ± 0	
Convex (n = 1)	51 ± 0		23 ± 0		52 ± 0	

a Bold indicates *P* < .05.

Increased glenoid retroversion (*P* = .004), posterior scapulohumeral decentering (*P* = .021) (Table 2), and dysplastic bony glenoid shape (*P* = .044) (Table 3) were associated with a worse 1-year durability of the treatment effect. This association could not be confirmed for the 2-year follow-up, however.

At the end of the intervention, the participants were very satisfied (81%) or satisfied (19%) with the shoulder-pacemaker treatment, and 100% would recommend it to others. No adverse events, except occasional muscle soreness, were observed during the treatment period or during follow-up.

## Discussion

Even though surgical treatment is effective for structural posterior shoulder instability, patients experiencing PP-FSI should not be treated surgically, since it often does not lead to the desired stabilization effect of the shoulder joint but instead to aggravated pain, limitation of shoulder function, and degenerative changes.^13,16,18,25,37^ Physical therapy as well as muscle activation training is generally recommended as the current treatment option of choice but has shown little success in several cases.^17,37^ In clinical practice, patients affected by PP-FSI sometimes visit several shoulder specialists and physical therapists, and after an extensive period of ineffective nonoperative therapy, they ultimately undergo a salvage surgical stabilization attempt with outcomes that can be worse than before surgery.^28,29^ Skillful neglect has been proposed as an alternative treatment option since symptoms may regress as patients get older over decades.^16,25^ However, this waiting approach seems undesirable for young and active patients who want to return to their active lifestyles as soon as possible. Ineffectiveness of treatment and failure to pinpoint the causative pathology with imaging sometimes leads to the false dismissal of PP-FSI as attention-seeking behavior or even a psychiatric condition.^14,28^ However, in a recent study, no mental health disorders were detectable in most patients experiencing FSI.^28^ In general, there is little agreement concerning the diagnosis, classification, and treatment of PP-FSI.

The characteristics of FSI with its subtype PP-FSI were recently described. In this pathology, nonphysiological muscle activation patterns seem to be the leading cause of instability rather than structural defects.^28^ Therefore, in this published cohort, the subgroup of patients experiencing noncontrollable PP-FSI was treated with an EMS-based therapy called the shoulder-pacemaker treatment concept in a prospective trial. Even though all patients had undergone conventional physical treatment for at least 3 months without success, a highly significant improvement of subjective and objective outcome parameters was achieved, and stability was reobtained in all participants within 3 to 6 weeks of treatment. Clinical improvement allowed most participants to resume their daily activities, and some patients even to return to high-demand shoulder sports (see the online Video Supplement). Follow-up results displayed a persistence of improvement over the course of 2 years. Only 3 cases showed a decrease of the treatment effect over time. In these cases, the noninvasive nature of the treatment allowed for repeat intervention in clinical practice. Since the treatment converts the previously noncontrollable condition into a controllable condition, the instability was still evocable by all patients at will after the intervention. Furthermore, occasional isolated subluxations can occur if the patient fails to concentrate and actively contract the muscle groups responsible for prevention of posterior subluxation. Similar observations were reported by Merolla et al^27^ for patients with voluntary posterior shoulder dislocations after receiving a dedicated rehabilitation program addressing scapular control and external rotators.

In general, the treatment goal in patients with PP-FSI is the correction of nonphysiological muscle activation patterns, abnormal motion, and poor body posture.^27^ Other groups attempted to achieve these goals by increasing patient awareness by means of careful explanation, as well as tactile, auditory, and visual biofeedback.^4,17,25,27,37,39,41^ Jaggi et al^18^ identified aberrant muscle patterns of the rotator cuff and periscapular muscles in an analysis of 131 atraumatic recurrent shoulder instability cases using dynamic fine-wire electromyography assessment and therefore applied resisted external rotation to prevent posterior instability during motion. Reinold et al^34^ performed EMS to strengthen peak shoulder external rotation force and minimize the inhibition of the infraspinatus after rotator cuff repair surgery. The presumed key to success of the shoulder-pacemaker treatment concept is similar, as it induces a contraction of external rotators and scapula-stabilizing retractors, which were previously hypoactive, and therefore stabilizes the shoulder. The effect of the treatment lasts over time even though the electrical stimulation is applied for only a short period. It seems that the EMS provides a “feed-forward” mechanism, meaning that patients perceive, realize, and learn which muscles they have to activate to stabilize their shoulders. Interestingly, young and more athletic patients with lower weight and unilateral pathology seem to accomplish this task more easily and respond better to the treatment. Cases with increased glenoid retroversion, posterior scapulohumeral decentering, and dysplastic bony glenoid shape showed a trend toward shorter longevity of the treatment results, probably because of the presence of structural deficiencies of the posterior glenoid on top of the functional deficiencies. Use of the shoulder-pacemaker treatment alongside surgical interventions in patients with combined structural and functional deficiencies needs to be evaluated scientifically.

### Limitations

A limitation of this study is the lack of a control group. However, the investigated cohort represents a negative selection since only patients with previous unsuccessful pathology-targeted physical therapy were included. Final follow-up assessment did not include a clinical examination in all patients. However, the main outcome measurements were subjective scores not requiring objective clinical examination, and video footage of instability-free motion was obtained from all patients. Furthermore, the data were gathered from only 1 institution, which introduces the risk for confirmation bias and limits generalizability of the results. In the case of bilateral PP-FSI, fluoroscopy and MRI of only the more severely affected side were obtained in an attempt to limit radiation exposure, duration of examination, and study costs. Even though the number of included cases seems low at first sight, it is, to our knowledge, the largest prospective and homogeneous collection of this rare pathology.

## Conclusion

The shoulder-pacemaker therapy concept is an effective treatment with rapid improvement and sustained outcome over the course of 2 years in patients experiencing noncontrollable PP-FSI with previously failed conventional treatment. Young and more athletic patients with lower weight and unilateral pathology responded best to the treatment. Structural deficiencies of the posterior glenoid might impair the longevity of the treatment effect.

## Supplemental Material

DS_10.1177_0363546520933841 – Supplemental material for Shoulder-Pacemaker Treatment Concept for Posterior Positional Functional Shoulder Instability: A Prospective Clinical Trial Click here for additional data file. Supplemental material, DS_10.1177_0363546520933841 for Shoulder-Pacemaker Treatment Concept for Posterior Positional Functional Shoulder Instability: A Prospective Clinical Trial by Philipp Moroder, Fabian Plachel, Heiko Van-Vliet, Christiane Adamczewski and Victor Danzinger in The American Journal of Sports Medicine
