# Supplementary material for: Shoulder-Pacemaker Treatment Concept for Posterior Positional Functional Shoulder Instability: A Prospective Clinical Trial
Source: Am J Sports Med. 2020 Jul 15;48(9):2097–104. doi: 10.1177/0363546520933841 (PMC7364790; doi:10.1177/0363546520933841)

Protocol of the Shoulder Pacemaker treatment concept for posterior positional functional shoulder instability

1. *Introduction*

Procedure:

- 3 sessions of treatment per week over a period of six weeks
- 1 session of treatment = 30 minutes electrical muscle stimulation exercises + 30 minutes regular exercises

Application of the electrical muscle stimulation (EMS) device

- The EMS device remains attached to the shoulder through the first 30 minutes of the physiotherapeutic treatment to stimulate hypoactive muscle groups during motion exercises (Phase 1).
- In the following 30 minutes the stimulated muscle groups are strengthened by physiotherapeutic exercises without electrical stimulation (Phase 2).
- Prior to the physiotherapeutic treatment, the intensity of the electric current is specified. It is important to use the highest current intensity the patient can comfortably handle (it typically increases over the course of the treatment).
- The electrode is positioned in the area of the nerve supplying the hypoactive muscles. Simultaneous stimulation of agonist and antagonist is to be avoided because of the resulting movement restriction. One electrode is placed inferior to the spina scapulae to stimulate the external rotators (M. infraspinatus, M. teres minor). The second electrode is placed medially to the margo medialis scapulae to stimulate the scapula retractors (M. trapezius pars transversa, Mm. rhomboidei).
- Aim for an initial abduction-external rotation of the arm due to the electrical stimulation of external rotators and scapula retractors. If sharp pain occurs slightly vary the electrode positioning or decrease the stimulation energy for comfortable training.

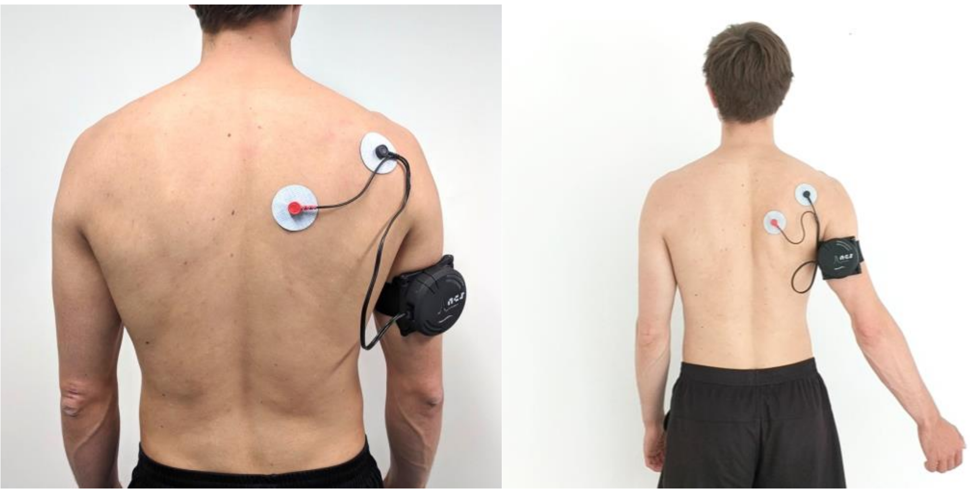

Example of the application of an EMS device (Shoulder Pacemaker™, NCS Lab, Carpi, Italy)

Phase 1: Exercises with electric stimulation under therapeutic supervision

- The initial movement exercises are divided into 3 segments:
  - Concentric exercises:
    Activation and tonic contraction of hypoactive muscle groups through maximum tolerable intensity of the EMS-device. Periodic change of stimulated contraction and subsequent relaxation in neutral position.
  - Eccentric exercises:
    Strengthening of hypoactive muscle groups by eccentric exercises using electrical stimulation. Periodic change of extension against the electrical stimulus and subsequent relaxation.
  - Functional exercises:
    Movement exercises with assisted electrical stimulation of hypoactive muscles are attempted to achieve a complete, instability-free movement of the shoulder joint. This can be achieved, for example, by patient-oriented sports training (e.g., boxing movements, throwing movements etc.).
  - In addition, patient-specific, iterative increase of the intensity and exercise complexity during the training (Level I-III).

Phase 2: Exercises without electric stimulation under therapeutic supervision

- Exercises are selected according to standard conventional physiotherapeutic concepts
- Successive increase of difficulty (Level I-III)
- Milestones:
  - Strengthening and coordination of the rotator cuff
  - Strengthening and coordination of the scapula-stabilizing muscles
  - Improvement of proprioception

2. Specific exercise program

Phase 1: Exercises with electric stimulation under therapeutic supervision

<u>Level I:</u>

- *Concentric exercises during* EMS-device *activation:*
  - Scapula Pinch:
    - Concentric training of the scapula retractors
    - Starting position: feet in line with the hip joints, arms in neutral position
    - Exercise: Bilateral scapula retraction, 3x15 repetitions with EMS-device stimulation on the affected side

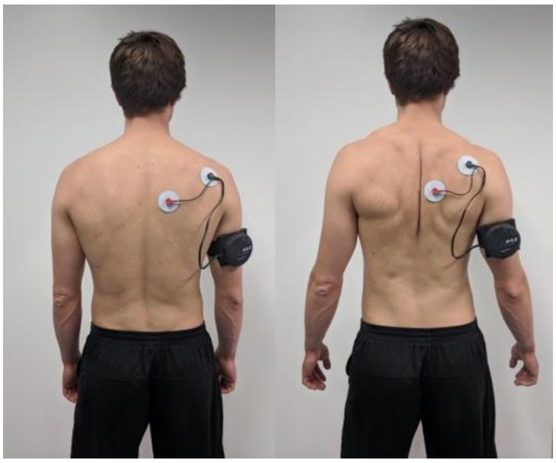

- *Eccentric exercises during* EMS-device *activation:*
  - Internal rotation:
    - Eccentric training of external rotators
    - Starting position: Arms in neutral position
    - Exercise: Internal rotation of the arms, back of the hands touching the lower back, 3x15 repetitions with EMS-device stimulation on the affected side

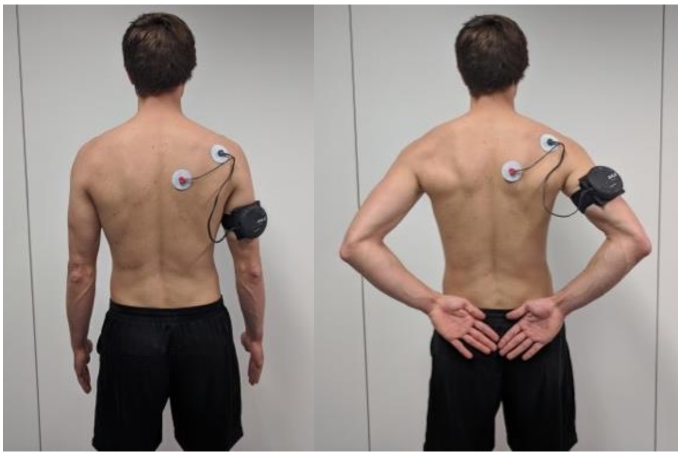

- *Functional exercises during* EMS-device *activation:*

  - Elevation with lateral resistance ("Lateral wall slide")
    - Starting position: Patient with the affected arm against a wall
    - Exercise: Elevation, back of the hand facing the wall, 3x15- 20 repetitions with EMS-device stimulation
    - Note: Start to 90° Flexion, slowly progress for pain free motion

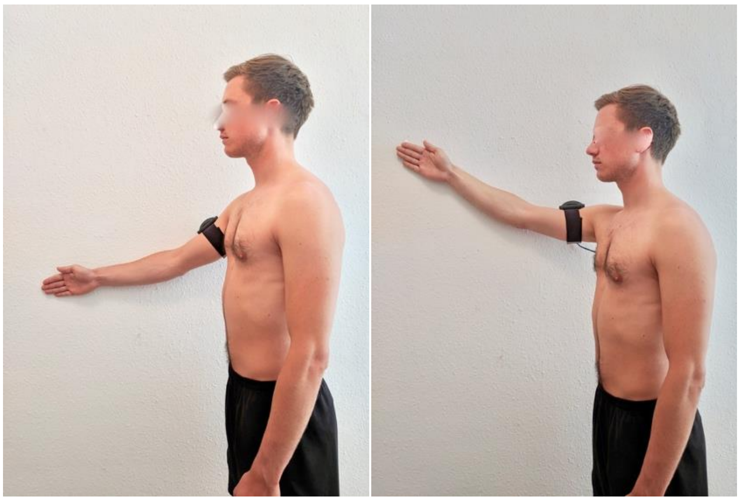

  - Wall Slide with resistance band
    - Starting position: Patient stands in front of the wall, feet in line with the hip joints, elbows flexed touching the wall, resistance band around both hands
    - Exercise: Slowly sliding the arms up the wall, 3x15- 20 repetitions with EMS-device stimulation
    - Note: Pain free motion, upright posture without increasing lumbar lordosis

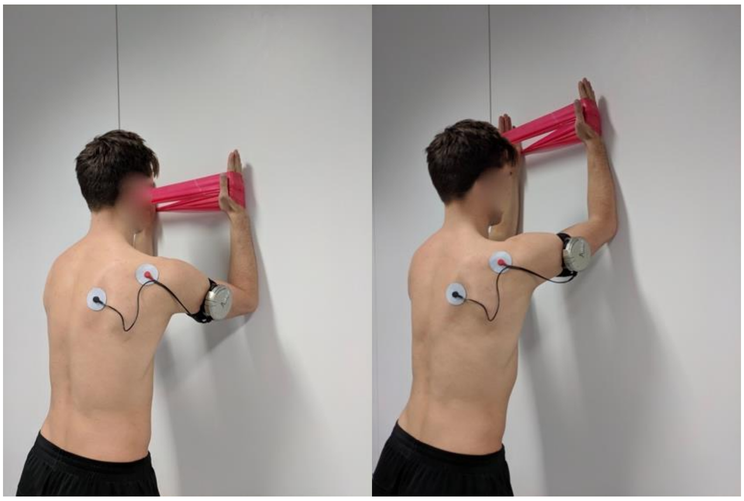

Level II:

- *Concentric exercises during* EMS-device *activation:*

  - External Rotation with trunk rotation:
    - Concentric training of external rotators
    - Starting position: Elbows in 90° flexion and hands neutral, shoulder in slight scaption position (towel/ball), resistance band in one hand
    - Exercise: External rotation against a resistance band, 3x15 repetitions with EMS-device stimulation

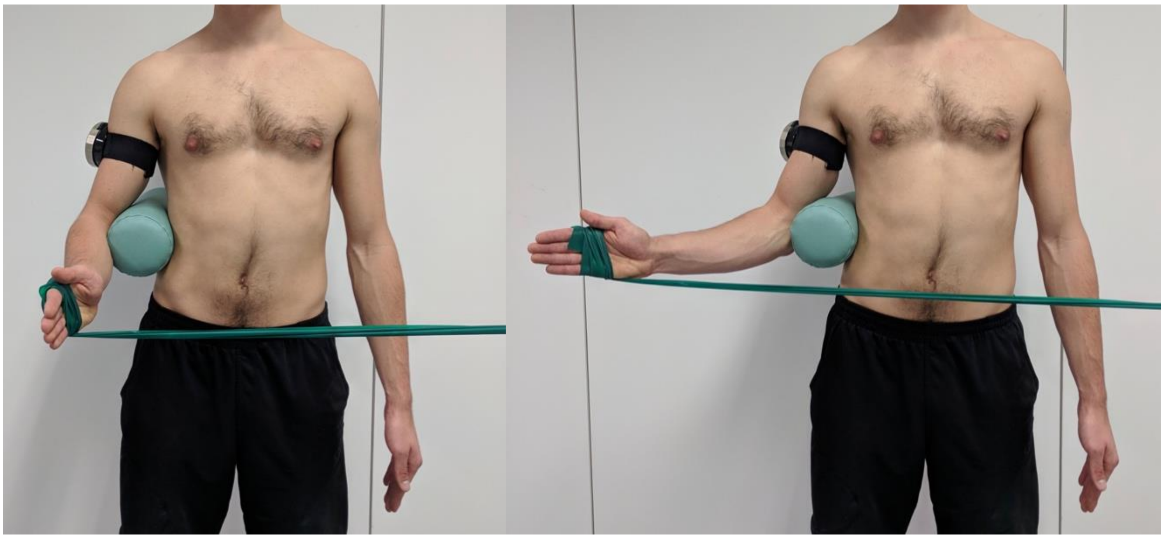

  - Standing One-handed Row:
    - Starting position: Elbows extended with one hand on a stable surface, resistance band in one hand with anchor at height of feet
    - Exercise: Pull and scapula retraction ("elbow in back pocket") against a resistance band with EMS-device stimulation, 3x15- 20 repetitions

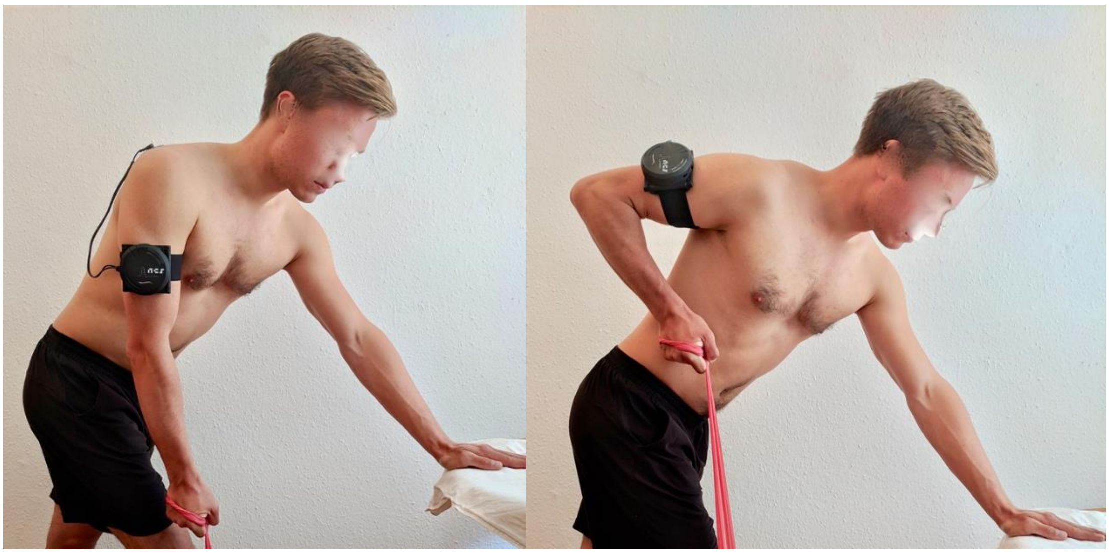

- *Eccentric exercises during* EMS-device *activation*:

  - Wall Push Up:
    - Eccentric training of scapula retractors
    - Starting position: Patient stands in front of the wall, feet in line with the hip joints, hand palms touching the wall, the scapulae are retracted
    - Exercise: Push-ups against the wall by means of shoulder protraction, 3x15 repetitions with EMS-device stimulation of the affected side
    - Cave: Elbows point towards the ground

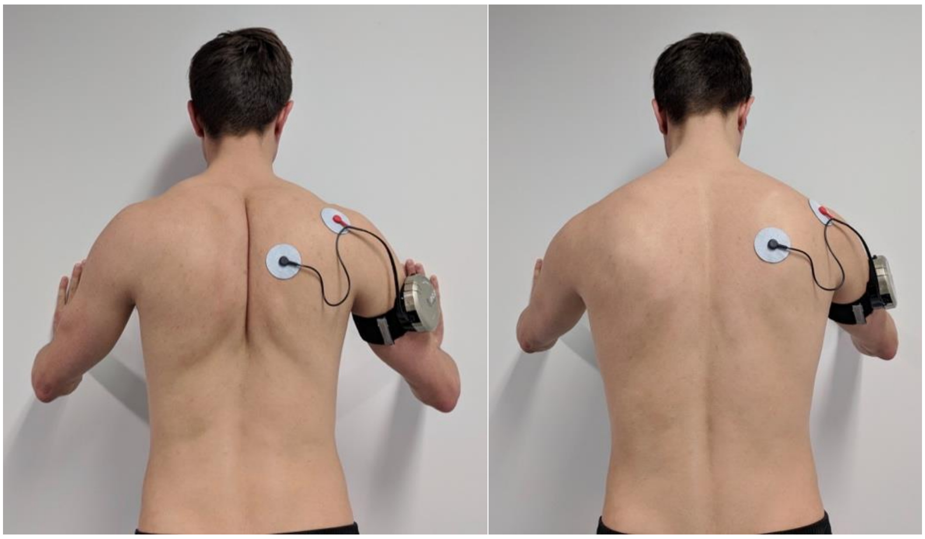

  - Horizontal Punches:
    - Starting position: Feet in line with the hip joints, elbow flexed
    - Exercise: Horizontal punches against the EMS-device induced contraction; 3x15 repetitions with EMS-device stimulation of the affected side

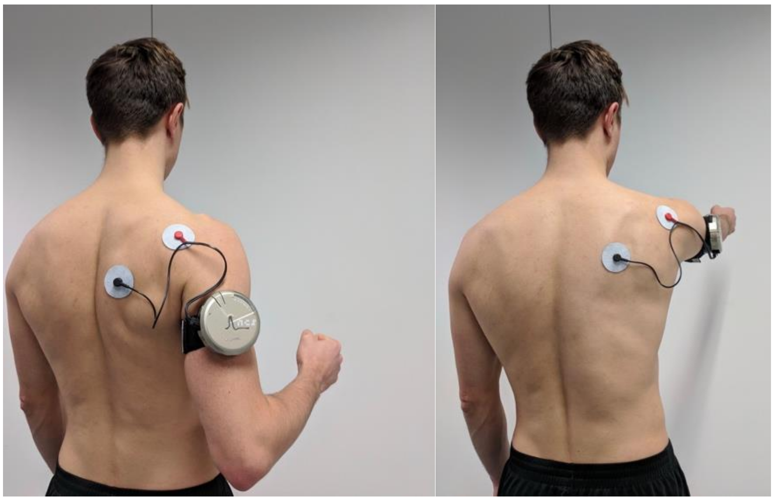

- *Functional exercises during* EMS-device *activation:*

  - Elevation against wall:
    - Elevation during stimulation of external rotators and scapula retractors and simultaneous external rotation against the resistance of a resistance band
    - Starting position: Knees and Hips slighly flexed, resistance band around both wrists and elbows flexed in 20° external rotation
    - Exercise: Instability-free elevation of both arms as high as possible, 3x15 repetitions with EMS-device stimulation on the affected side

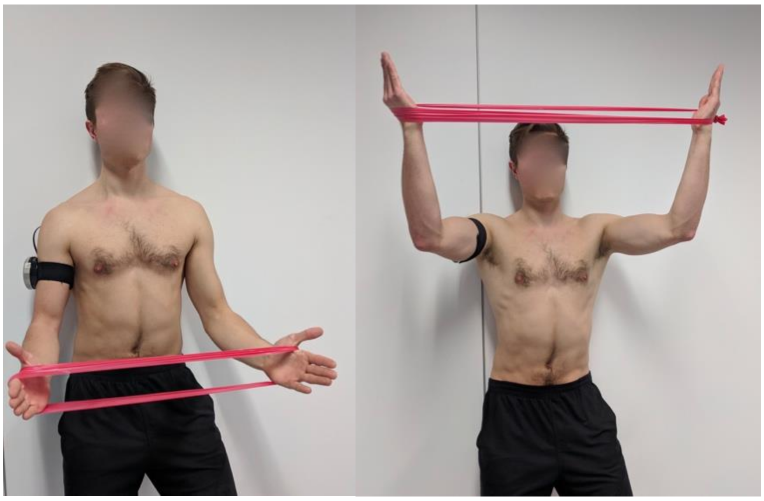

  - Elevation and Abduction:
    - Free elevation and abduction during stimulation of external rotators and scapula retractors
    - Starting position: Neutral position
    - Exercise: Instability-free elevation and abduction of both arms as high as possible, 3x15 repetitions with EMS-device stimulation of the affected side

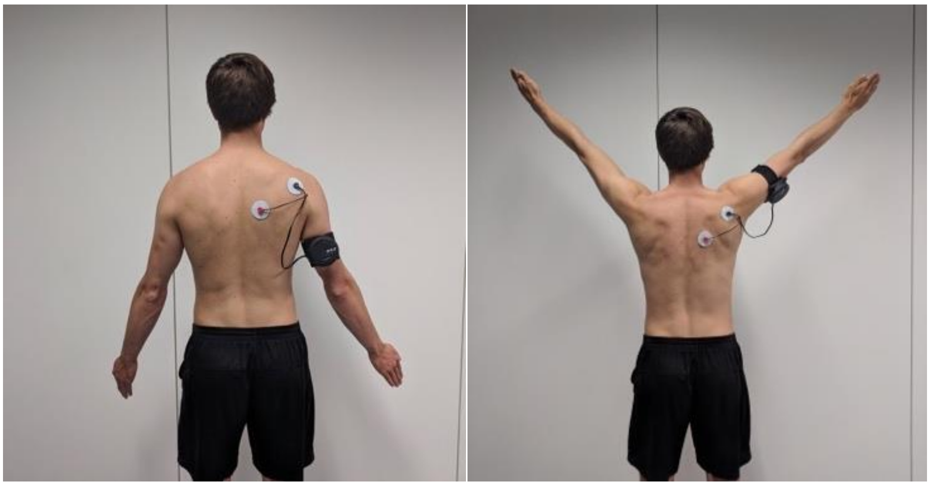

- Patient Oriented Sports Training (POST):
  - Movement exercises *during* EMS-device *activation* (e.g. throwing, boxing)

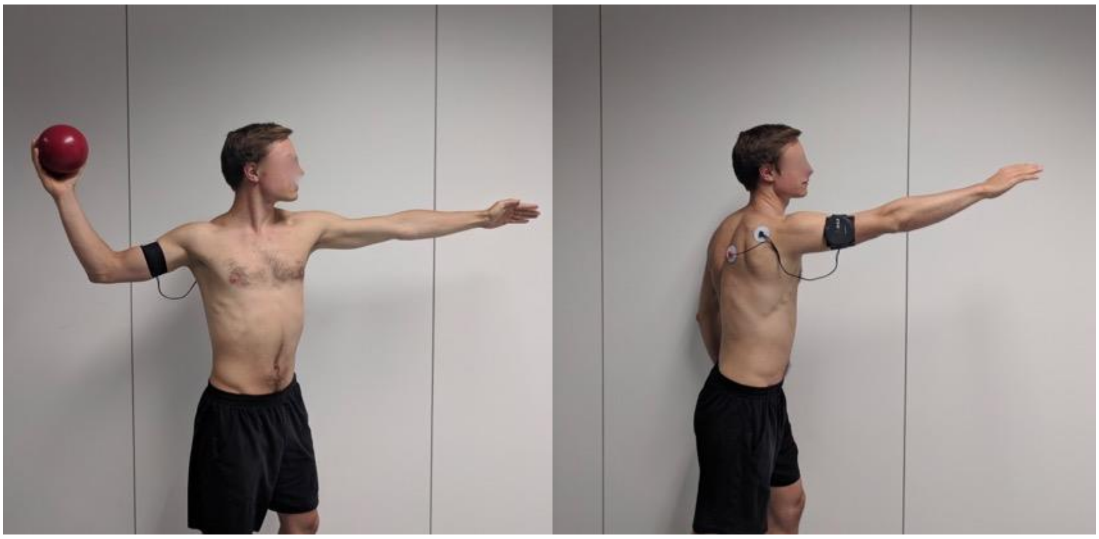

Level III:

- *Concentric exercises during* EMS-device *activation*:

    - Reverse Butterfly:
        - Concentric training of scapula retractors
        - Starting position: Arms in 90° abduction und 30° horizontal-flexion, elbows extended, thumbs point upwards, resistance band in both hands
        - Exercise: Bilateral horizontal extension of both arms and scapula retraction against a resistance band, 3x15 repetitions with EMS-device stimulation of the affected side

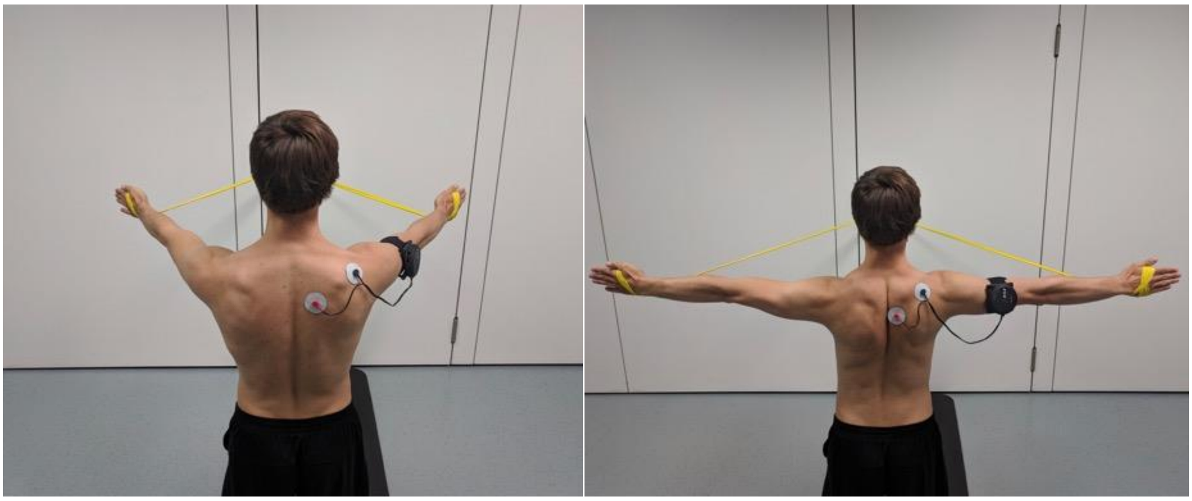

    - Concentric low row
        - Starting position: feet positioned in line with hip joints, hips/trunk flexed, resistance band in both hands with anchor at height of feet
        - Exercise: Concentric low row motion with hips/trunk extension and scapula retraction with EMS-device stimulation, 3x15-20 repetitions

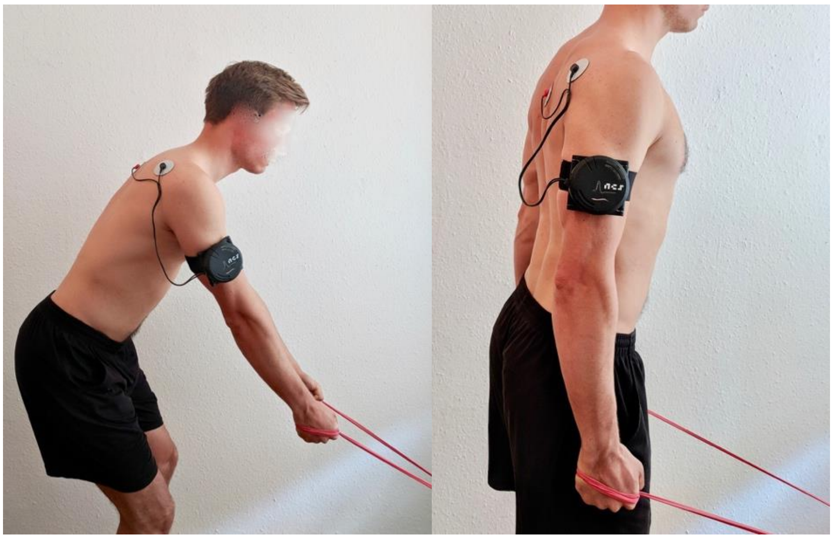

- *Eccentric exercises during* EMS-device *activation:*

  - Push Up Plus:
    - Eccentric training of scapula retractors
    - Starting position: Push-up position, palms in line with the shoulder joints
    - Exercise: Push-ups with scapula protraction, 3x15 repetitions with EMS-device stimulation of the affected side
    - Cave: Elbows point towards the floor

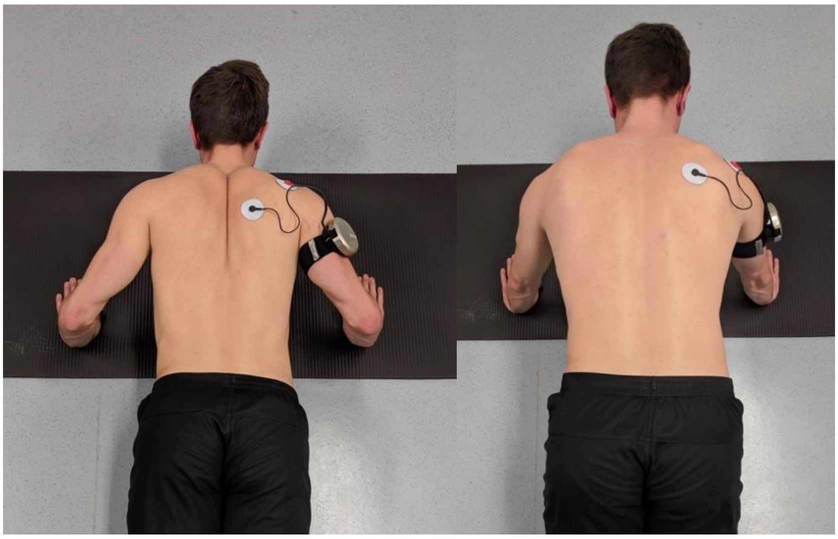

  - Horizontal Punch Plus:
    - Starting position: Feet in line with hip joints, elbows slightly flexed, resistance band in one hand
    - Exercise: Horizontal punches against a resistance band, 3x15 repetitions with EMS-device stimulation of the affected side

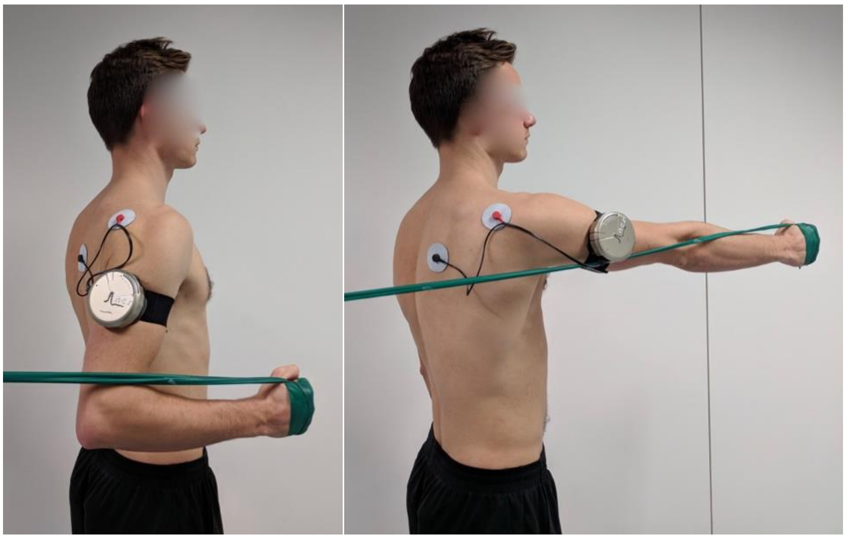

- *Functional exercises under the application of the* EMS-device*:*

  - Elevation with a resistance band:
    - Elevation during stimulation of external rotators and scapula retractors and simultaneous external rotation against the resistance of a resistance band
    - Starting position: Hips and knees flexed, resistance band around both hands
    - Exercise: Instability-free elevation of both arms, 3x15 repetitions with EMS-device stimulation on the affected side

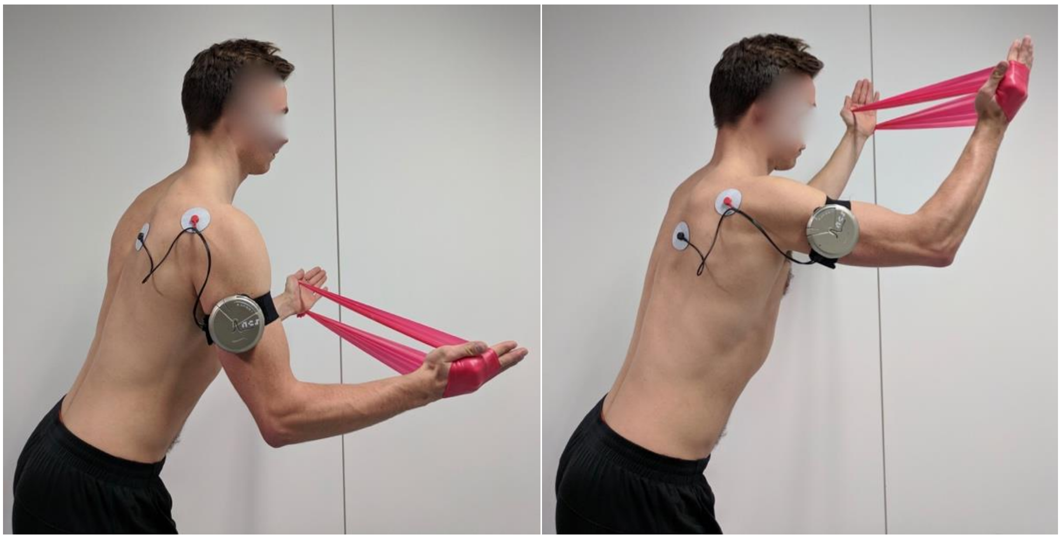

  - Elevation and Abduction:
    - Free elevation and abduction during stimulation of external rotators and scapula retractors
    - Starting position: Neutral position
    - Exercise: Instability-free elevation and abduction of both arms as high as possible, 3x15 repetitions with EMS-device stimulation of the affected side

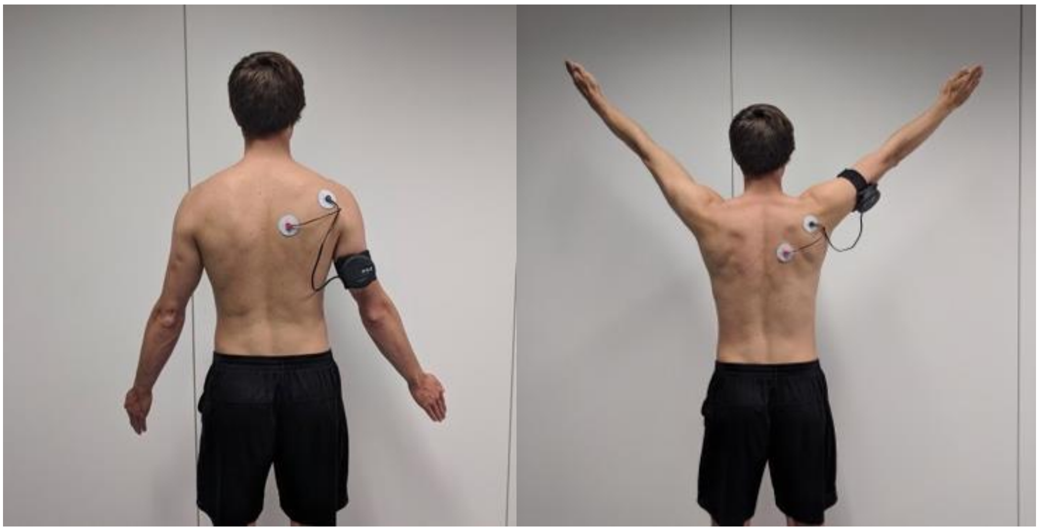

- POST Plus:
  - Movement exercises *during* EMS-device *activation* (e.g. throwing, boxing) with increased intensity (repetitions, complexity)

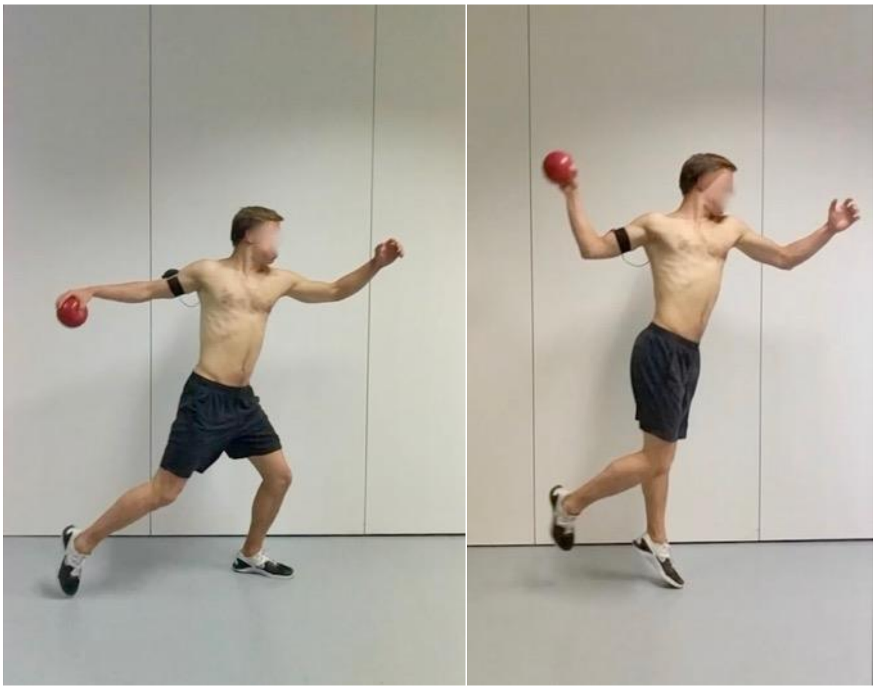

Phase 2: Exercises without electric stimulation under therapeutic supervision

Exercise 1: Prone retroflexion ("I")

- Activation of the scapula stabilizers
- Starting position: prone position, elbows extended, thumbs in external rotation
- Exercise: Retroflexion and scapula retraction holding fully retracted position for 2-5 seconds, 3x15-20 repetitions

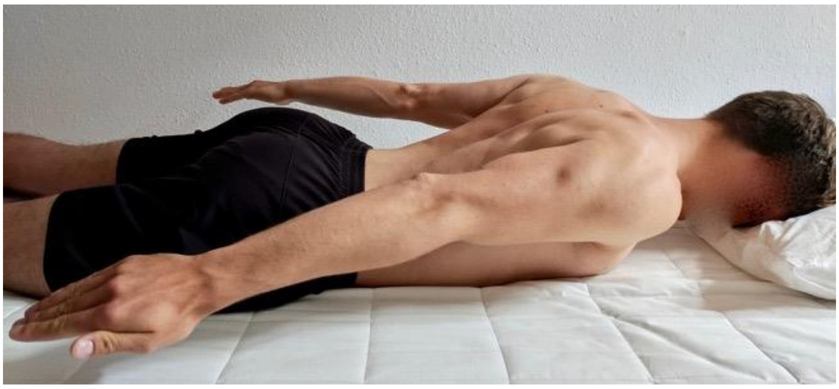

Exercise 2: Prone horizontal external rotation ("L")

- Activation of the scapula stabilizers
- Starting position: prone position, shoulder in 90° flexion and abduction, elbows 90° flexed, thumbs points upwards in external rotation
- Exercise: External rotation and scapula retraction holding fully retracted position for 2-5 seconds, 3x15-20 repetitions

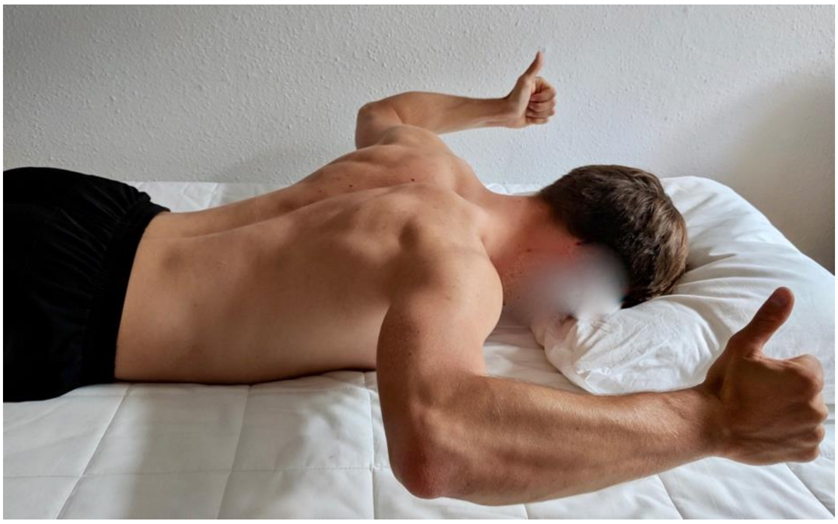

Exercise 3: Prone horizontal abduction and external rotation ("T")

- Activation of the scapula stabilizers
- Starting position: prone position, shoulder in 90° flexion and abduction, elbows extended, thumbs points upwards in external rotation
- Exercise: Raise arms and scapula retraction holding fully retracted position for 2-5 seconds, 3x15-20 repetitions

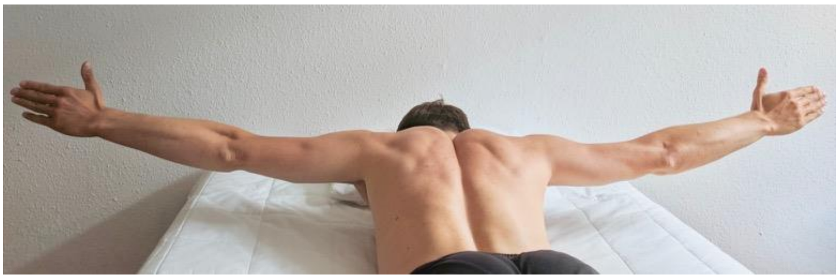

Exercise 4: Ball Wall Circles

- Starting position: Patient holds a ball in the hand of the affected arm and leans against a wall in 90° abduction
- Exercise: Lean body towards the wall whilst stabilizing the position of the hand on the ball, perform small circle motions, 3x15- 20 repetitions

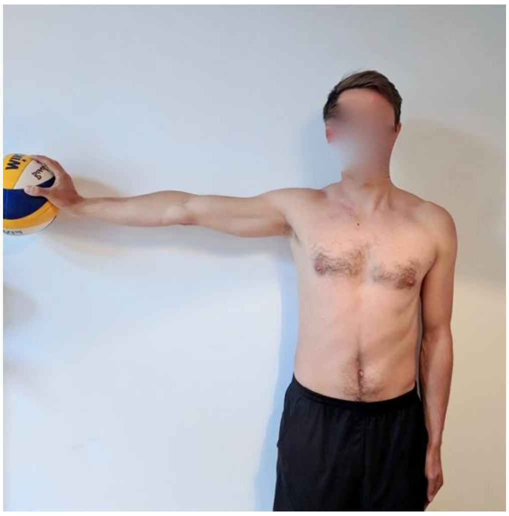

Exercise 5: Ball Wall Stabilization

- Starting position: Patient stabilizes with elbows flexed on a ball and leans against a wall
- Exercise: Stabilizing the position on the ball (5x 20 sec.)
- Cave: Keep shoulders stable

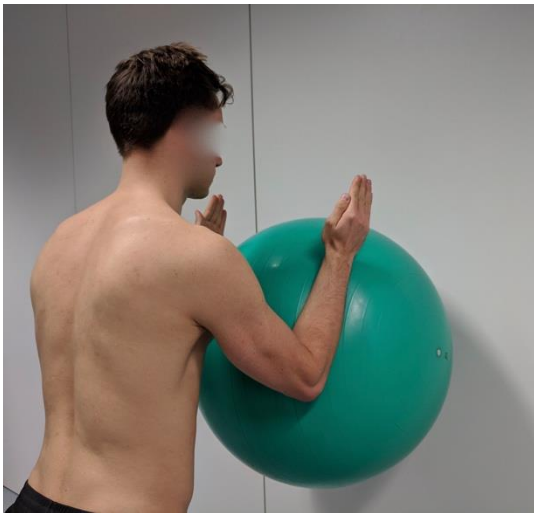

Supplement: DS_10.1177_0363546520933841 – Supplemental material for Shoulder-Pacemaker Treatment Concept for Posterior Positional Functional Shoulder Instability: A Prospective Clinical Trial [file DS_10.1177_0363546520933841.pdf]
